# Commissioning a ‘neighbourhood health service’: what can we learn from the literature?

**DOI:** 10.3399/BJGP.2025.0177

**Published:** 2025-10-27

**Authors:** Kath Checkland, Donna Bramwell, Simon Bailey, Karen Proctor, Jonathan Hammond, Lynsey Warwick-Giles

**Affiliations:** 1 Division of Population Health, Health Services Research and Primary Care, University of Manchester, Manchester, UK; 2 Centre for Health Services Studies, University of Kent, Kent, UK; 3 University of Manchester, Manchester, UK

## Introduction

The recently published NHS 10 Year Health Plan^1^ envisages an NHS built around *‘neighbourhood health services’*, with a significant rebalancing of investment and spending away from hospitals to services delivered in local communities. The plan is clear that each area will be free to develop its own service model, but a number of elements are highlighted as being important. These include:

- neighbourhood health teams, providing integrated care across defined geographical areas;
- better GP access, including digital access and the potential for more primary care to be delivered at scale;
- care plans for those with multiple long-term conditions;
- expanded access to personal health budgets; and
- new neighbourhood health entres, housing a range of services in a single building.

Many of these things currently exist in many areas, but the plan highlights the need for a more consistent and comprehensive approach across England. In delivering these things, the plan sets out a structure that includes a leaner and less directive centre (bringing together the functions of the Department of Health and Social Care and NHS England), seven regional teams, and a smaller number of reconfigured Integrated Care Boards (ICBs), with their geographical footprints adjusted as necessary to better match local authority boundaries. Importantly, the role of ICBs as commissioners of care is reiterated, with a renewed focus on taking a strategic approach to commissioning care from a market of providers and with boards reconfigured to remove provider representatives. The plan also references the creation of new forms of contract, by which ICBs may contract with either a single neighbourhood provider (assumed to correspond to the footprint of a single Primary Care Network) or a multiple neighbourhood provider to deliver the required neighbourhood services.

There is clearly much to be clarified about the components of the envisaged neighbourhood care, as well as their delivery in practice. However, our focus in this article is upon *the commissioning process*. Given the strong focus within the plan upon the role of ICBs in shaping the market of local neighbourhood providers, we provide an evidence-informed commentary on what infrastructure and local processes are likely to be necessary to support the changes. We argue that, without attention to who sets local goals, how commissioning decisions are made, available mechanisms for allocating resources, and approaches to oversight and monitoring, the difficult task of breaking down barriers between sectors and organisations cannot be achieved. Without effective commissioning infrastructure it is unlikely that the new service models will be effectively delivered.

To explore what might be required to commission a ‘neighbourhood health service’, we examined two broad literatures: evidence related to commissioning; and the evidence around the implementation of integrated care initiatives. This was not a systematic review; rather, in both literatures we focused upon significant review articles to answer the question: what local processes and structures are required if integrated care systems are to effectively commission integrated neighbourhood services?

## The Health and Care Act 2022

The Health and Care Act 2022 (HCA2022) abolished around 190 clinical commissioning groups (CCGs), replacing them with 42 integrated care systems (ICSs), responsible for health services for populations of around 1–3 million people. While the act downgraded the importance of competition and emphasised collaboration across the ICS footprint, the HCA2022 left intact the underlying system architecture. Thus, statutory responsibilities that previously sat with CCGs were transferred to the ICB — the ICS executive group initially made up of senior representatives of local providers and other system partner organisations. Notwithstanding the need to collaborate, the underlying mechanisms by which services are provided remained the same, with the ICB responsible for resource allocation and provider contracting. ICBs cover large geographical areas (with several now to be merged to cover even larger footprints), and guidance suggests that responsibility for most decision making about local services should be devolved to ‘places’ (roughly corresponding to previous CCG footprints or existing local authority areas), with delivery optimised across ‘neighbourhoods’.^2^ These are not formally designated, but generally assumed to represent footprints across which local services such as general practices and Community Nursing Services work together.^2^ Thus, although ‘neighbourhood health service’ was not initially clearly defined, its delivery is likely to entail planning and decision making at place level.

## What is commissioning?

Commissioning derives from Øvretvreit’s purchasing framework,^3^ which describes the cyclical process of strategic service planning including needs assessment, planning, contracting, monitoring, and review. The resulting ‘commissioning cycle’ has underpinned service development in the NHS in England for many years.^4^ A considerable body of research on commissioning exists.^5–11^ A recurring theme is the extent to which transactional approaches common in the private sector — whereby marketisation supports frequent shifting of contracted provision — are inappropriate within a publicly funded service where market failure would be disastrous. This literature highlights the importance of a relational approach, where commissioners and providers work together to optimise services and health outcomes.^12^

Wade *et al* ^13^ studied commissioning by primary care trusts. While NHS structures have changed, the underlying architecture of service planning remains the same, and these activities therefore remain relevant:

- objective setting and decision making, including: balance between national/regional/local objectives; mechanisms for setting local objectives; clarity over scope of decision-making powers; and governance structures by which they can be held to account;
- facilitation and management of partnerships across their geographical footprint;
- information collection and analysis, including: population health needs; service maps; provider activity and quality data; patient satisfaction data; and intelligence about factors affecting demand;
- patient and public engagement to ensure population needs and preferences are properly understood;
- service design and resource allocation, with some more specialised services designed and delivered over larger footprints by consortia of commissioners; and
- procurement and contracting, including: service specifications that take account of evidence as well as local service provider capacity and capability; contracting procedures; contract monitoring; quality improvement; and performance management.

In addition, reviews have highlighted the fact that the geographical scale over which commissioning should take place depends upon a trade-off between the need for local knowledge, relationships, and responsiveness to fine-grained assessments of need, and the requirement for risk sharing and increased commissioner power for more specialised services or larger-scale reconfigurations.^10,14,15^

## Integrated service delivery

‘Integrated care’ is often poorly defined, with one article identifying more than 175 overlapping definitions.^16^ The NHS Long Term Plan^17^ references *‘more joined-up and coordinated … care’* and *‘*[b]*reaking down traditional barriers between care institutions, teams and funding streams’*, while an initial ICS design document highlighted the need to: *‘join up planning and service delivery across historical divides: primary and specialist care, physical and mental health, health and social care’*.^18^ A review of integration initiatives in the NHS^19^ highlighted the ambiguity at the heart of many ‘integration’ initiatives, concluding that efforts to improve patient experiences should focus upon care coordination across organisational, professional, and geographical boundaries. It is this conceptualisation of integration that is foremost in the 10 Year Health Plan, with a strong focus on *‘a single, coordinated, patient-orientated service’*.^1^

**Figure 1.**
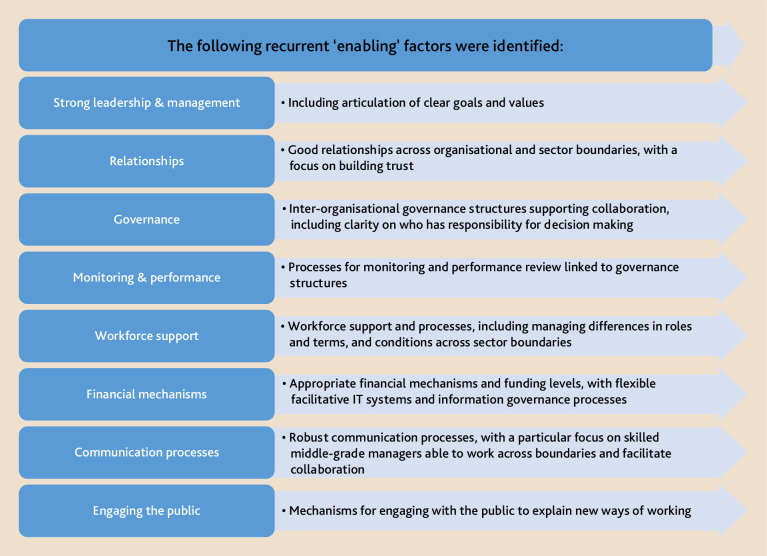
Factors enabling the provision of integrated services

We explored review articles and published evaluations of national and international integration initiatives, focusing upon those involving integration across organisational and sector boundaries (Table 1; Supplementary Data S1). Recurrent ‘enabling’ factors were identified as per Figure 1.

**Table 1.** Included articles

Author	Date	Country	Title
Baxter *et al*	2018	UK & International	The effects of integrated care: a systematic review of UK and international evidence
Bhat *et al*	2022	UK	Identifying and understanding the factors that influence the functioning of integrated care in the NHS: a systematic literature review
Cameron *et al*	2014	UK	Factors that promote and hinder joint and integrated working between health and social care services: a review of research literature
Checkland *et al*	2022	UK	*National Evaluation of the Vanguard New Care Models Programme: Final Report*. Manchester: University of Manchester
Erens *et al*	2015	UK	*Early Evaluation of the Integrated Care and Support Pioneers Programme: Final Report*. London: Policy Innovation Research Unit
Goodwin & Smith	2011	UK	*The Evidence Base for Integrated Care*. London: King’s Fund
Kelly *et al*	2020	UK & International	Measures for the integration of health and social care services for long-term health conditions: a systematic review of reviews
Kirst *et al*	2017	UK & International	What works in implementation of integrated care programs for older adults with complex needs? A realist review
Leitjen *et al*	2018	UK & International	The SELFIE framework for integrated care for multi-morbidity: development and description
Lewis *et al*	2021	UK	Integrated care in England — what can we learn from a decade of national pilot programmes
Miller *et al*	2021	UK	Integrated health and social care in England: ten years on
Neiva *et al*	2023	Brazil	How is integration defined and measured, and what factors drive success in Brazil? An integrative review
Piquer-Martinez *et al*	2024	UK & International	Theories, models and frameworks for health systems integration. A scoping review
Round *et al*	2018	UK	An integrated care programme in London: qualitative evaluation
Steele Gray *et al*	2020	Canada, New Zealand, the Netherlands	Comparing international models of integrated care: how can we learn across borders?
Thomson & Chatterjee	2024	UK	Barriers and enablers of integrated care in the UK: a rapid evidence review of review articles and grey literature 2018–2022

Overall, the international literature suggests clarity over roles and responsibilities as well as the vision and goals underpinning integration initiatives are important. Trust across organisational boundaries is vital, requiring active mechanisms and skilled managers to build and maintain it.

## Synthesis: commissioning integrated services in neighbourhoods

Bringing these literatures together allows exploration of what might be required if ICSs are to successfully commission more integrated services across neighbourhoods.

First, while talking about formal structures and decision-making processes can be perceived as overly managerial and lacking in collaborative spirit, the international literature on integration clearly highlights the importance of clear goals and decision-making processes. This means that there needs to be clarity around who is responsible for commissioning neighbourhood health services, and those responsible need to understand the scope of their decision-making powers. Importantly, the commissioning literature highlights the fact that it is important that those undertaking this role have close and trusting relationships with local service providers, as commissioning services is essentially a relational activity. This is particularly important when trying to support providers in working together across organisational and sector boundaries, as this can be difficult and may require expert facilitation and brokerage alongside the more formal apparatus of contract management. The commissioning literature suggests that this type of day-to-day activity needs to take place over relatively small geographical footprints to allow these relationships to develop and be effectively managed.^14,20^

Second, evidence relating to both commissioning and the provision of integrated services suggests that, while leaders with vision are needed, so are experienced managers with skills to manage relationships and the authority to broker agreements where organisational goals differ — over-riding individual organisational interests where necessary.

Third, evidence suggests that there needs to be a comprehensive approach to population health data, with streamlined mechanisms for sharing across organisational boundaries and staff with relevant analytical skills to transform that data into meaningful insights.

Finally, clarity around budget allocation is needed, and financial and procurement rules must facilitate rather than impede collaboration. These need to go along with mechanisms for monitoring activity and quality of services, and some formal body needs authority to intervene if outcomes do not accord with goals. There also needs to be appropriate balance between local and national priorities, without excessive national control of the agenda. This is important, because it is in developing and working towards collectively defined goals that trusting relationships will thrive.

Thus, the evidence suggests that the delivery of integrated neighbourhood services requires active commissioning by knowledgeable and trusted local commissioning managers, with access to appropriate data analysis and quality monitoring expertise, who need to work over a geographical scale small enough to allow relationships to develop and services to be tailored to local population needs.

## What does the 10 Year Health Plan say about commissioning neighbourhood health services?

The chapter of the 10 Year Health Plan^1^ that focuses upon NHS structures highlights the importance of active commissioning, and positions the new, larger ICBs as the default commissioners of neighbourhood health services. This raises questions about the appropriate scale over which such activity should occur. ICBs are much larger than we have seen for NHS commissioning organisations in the past, and they are set to get larger, with average population size increasing to nearer 3 million people. Both primary care trusts and clinical commissioning groups commissioned services for populations of, on average, around 300 000 — what we would now call ‘place’. The guidance surrounding the implementation of the act recognised the need for local commissioning activity, suggesting that the norm should be for day-to-day commissioning decisions to be delegated to place-level structures, although discretion was allowed as to how this was achieved.^21,22^ In practice little such delegation has occurred,^23^ largely because of the need to maintain tight financial control across the ICB footprint and the perception that places do not have the formal structures necessary to do this.^23^ However, this lack of formal structures is, in part at least, the result of decision making by those same ICBs, suggesting that it could be changed if there were sufficient will and appropriate incentives to support delegation of responsibilities.

### Conclusion

Our survey of relevant literatures suggests the desired shift towards delivering a neighbourhood health service will require the establishment of formal commissioning structures at place level, with local commissioners empowered to make the decisions needed to tailor services to population need, while at the same time engineering appropriate accountability and risk management structures. This could quickly be achieved by establishing formal commissioning committees within places, although it is also possible that other models (such as commissioning by a single provider holding a population budget) may be feasible.^14^ Whichever commissioning structure is chosen, evidence shows that skilled and responsive management of relationships between different organisations and sectors requires empowered managers who understand the scope of their role and the needs and preferences of their local populations. The literature on integration suggests that, while some functions such as data analysis can be done at a higher level, for most aspects of commissioning top-down management by large-scale ICBs will not deliver what is required. We would argue that that the establishment of effective place-level commissioning structures to support local collaboration is as important as deciding which services need to be delivered.

## References

[1] Department of Health and Social Care (2025.). Fit for the future: 10 Year Health Plan for England.

[2] NHS England (2022.). Next steps for integrating primary care: Fuller stocktake report.

[3] Øvretveit J (1993). Purchasing for health gain: the problems and prospects for purchasing for health gain in the ‘managed markets’ of the NHS and other European health systems. Eur J Public Health.

[4] Department of Health (2007.). World class commissioning: vision.

[5] Baxter K, Weiss M, Le Grand J (2008). The dynamics of commissioning across organisational and clinical boundaries. J Health Organ Manag.

[6] Bovaird T, Briggs I, Willis M (2014). Strategic commissioning in the UK: service improvement cycle or just going round in circles?. Local Government Studies.

[7] Bravo Vergel Y, Ferguson B (2006). Difficult commissioning choices: lessons from English primary care trusts. J Health Serv Res Policy.

[8] Chappel D, Miller P, Parkin D, Thomson R (1999). Models of commissioning health services in the British National Health Service: a literature review. J Public Health (Oxf).

[9] Checkland K, Harrison S, Snow S (2012). Commissioning in the English National Health Service: what’s the problem?. J Soc Pol.

[10] Curry N, Goodwin N, Naylor C, Robertson R (2008). Practice-based commissioning: reinvigorate, replace or abandon.

[11] Davies A (2007). A tangled web? Accountability and the commissioning role in the ‘new’ NHS. Kings Law J.

[12] Allen P (2002). A socio-legal and economic analysis of contracting in the NHS internal market using a case study of contracting for district nursing. Soc Sci Med.

[13] Wade E, Smith J, Peck E, Freeman T (2006). Commissioning in the reformed NHS: policy into practice. https://citeseerx.ist.psu.edu/document?repid=rep1&type=pdf&doi=505bafeeb8137e18c2ea30e5b9b74a6374bdd9a9.

[14] Smith J, Curry N, Mays N, Dixon J (2010). Where next for commissioning in the English NHS.

[15] Sheaff R, Chambers N, Charles N (2013). How managed a market? Modes of commissioning in England and Germany. BMC Health Serv Res.

[16] Armitage GD, Suter E, Oelke ND, Adair CE (2009). Health systems integration: state of the evidence. Int J Integr Care.

[17] NHS England (2019.). The NHS long term plan.

[18] NHS England (2019.). Designing integrated care systems (ICSs) in England.

[19] Lewis RQ, Checkland K, Durand MA (2021). Integrated care in england - what can we learn from a decade of national pilot programmes?. Int J Integr Care.

[20] Checkland K (2020). Ideal size of commissioning organisation — briefing note. https://pru.hssc.ac.uk/outputs/reports/ideal-size-of-commissioning-organisation-%E2%80%93-briefing-note.html.

[21] NHS England (2021). Integrated care systems: design framework. https://www.england.nhs.uk/wp-content/uploads/2021/06/B0642-ics-design-framework-june-2021.pdf.

[22] NHS England (2021). Thriving places: guidance on the development of place-based partnerships as part of statutory integrated care systems. https://www.england.nhs.uk/wp-content/uploads/2021/06/B0660-ics-implementation-guidance-on-thriving-places.pdf.

[23] Sanderson M, Allen P, Osipovic D (2022). The developing architecture of system management: integrated care systems and sustainability and transformation partnerships. https://pru.hssc.ac.uk/assets/uploads/files/the-developing-architecture-of-system-management-integrated-care.pdf.

